# Hepatoprotective, Antioxidant, and Anticancer Effects of the *Tragopogon porrifolius* Methanolic Extract

**DOI:** 10.1155/2015/161720

**Published:** 2015-01-28

**Authors:** Clara Tenkerian, Mirvat El-Sibai, Costantine F. Daher, Mohamad Mroueh

**Affiliations:** ^1^Department of Natural Sciences, School of Arts and Sciences, Lebanese American University, P.O. Box 36, Byblos 1401, Lebanon; ^2^Department of Pharmaceutical Sciences, School of Pharmacy, Lebanese American University, P.O. Box 36, Byblos 1401, Lebanon

## Abstract

*Tragopogon porrifolius* (Asteraceae), commonly referred to as white salsify, is an edible herb used in Lebanese folk medicine to treat cancer and liver dysfunction. In this study, we investigated the antioxidant activity of *Tragopogon porrifolius* methanolic extract, both *in vitro* and *in vivo*, in addition to its hepatoprotective and anticancer activities. Total phenolic and flavonoid contents were measured and found to be 37.0 ± 1.40 mg GAE/g and 16.6 ± 0.42 mg QE/g dry weight, respectively. *In vitro* antioxidant assays revealed an FRAP value of 659 ± 13.8 *µ*mol Fe^2+^/g of extract and DPPH IC_50_ value 15.2 *µ*g/mL. In rats subjected to CCl_4_-induced hepatotoxicity, significant increase in CAT, SOD, and GST levels was detected. The highest dose of the extract (250 mg/kg) recorded a fold increase of 1.68 for SOD, 2.49 for GST, and 3.2 for CAT. The extract also showed substantial decrease in AST (57%), ALT (56%), and LDH (65%) levels. Additionally, the extract caused a dose-dependent decrease in cell viability and proliferation. In conclusion, the methanolic extract of *T. porrifolius* displayed a relatively high antioxidant activity both *in vitro* and *in vivo* as well as hepatoprotective potential against liver toxicity in rats and anticancer effect on MDA-MB-231 and Caco-2 cells.

## 1. Introduction

Oxygen free radicals or reactive oxygen species (ROS), including the superoxide radical O_2_^•−^, hydrogen peroxide H_2_O_2_, and the highly reactive hydroxyl radical ^•^OH, are extremely reactive molecules and can oxidize lipids, proteins, and even DNA. They are normally present in a balance with antioxidants molecules that improve the body's cellular defense system against oxidative damage. Antioxidants help maintain lower levels of free radicals; thus they perform beneficial physiological roles [1, 2]. The antioxidant defense system of the body can be in the form of low molecular weight antioxidants such as vitamins E and C which block free radicals, or in the form of enzymes such as superoxide dismutase, catalase, and the glutathione system (glutathione, glutathione reductase, peroxidase, and transferase) that reduce the levels of reactive oxygen species [3, 4]. When an imbalance occurs between free radicals and the antioxidant defense system, this may result in oxidative stress, which has been shown to be involved in the initiation and progression of various human diseases, including cancer [2, 3]. DNA damage induced by both exogenous and endogenous free radicals is well documented and widely accepted to be a major cause of genomic instability and cancer. Human studies have shown that oxidative DNA damage is an important carcinogenic and mutagenic factor in the sense that it favors the acquisition of mutations and contributes to cellular transformation and cancer cell survival [2].

According to the World Health Organization [5], 80% of the population of developing countries in Asia and Africa rely on traditional medicine for primary health care. Resorting to traditional medicine in order to discover plants with therapeutic properties has proven valuable in the search for new bioactive compounds [6]. In fact, in recent years, concerns over harmful side effects of synthetic compounds have shifted the focus to natural plant resources, which represent an abundant source of biologically active molecules [7]. Taking into account that only 1% of the estimated 500,000 plant species on Earth have been investigated, the need for novel medicinal bioactive compounds is substantial [7].

*Tragopogon porrifolius*, Asteraceae family, is an edible herb and is commonly known as salsify, oyster plant, and vegetable oyster.* T. porrifolius* is widespread throughout the Mediterranean region where it grows wild and is cultivated. All parts of the plant are edible, the roots, leafy shoots, and open flowers are used as being both cooked and raw [8], and, in Lebanon, the shoots are more frequently consumed than the roots. The nutritional value of this plant has been attributed to its monounsaturated and essential fatty acids, vitamins, polyphenols, and fructooligosaccharides components [9]. Recent studies in our laboratory revealed that intake of aqueous extract of* T. porrifolius* caused improvement of lipemia and increased satiety in rats with no visible adverse effects [10]. Additionally, methanol, ethyl acetate, and chloroform extracts of aerial parts of* T. porrifolius* demonstrated anti-inflammatory effects in mice [11].

To the best of our knowledge, no studies were conducted on* T. porrifolius* to investigate its antioxidant, hepatoprotective, and anticancer activities. Therefore, this study was carried out to evaluate the* in vitro* and* in vivo* antioxidant activities of the plant methanolic extract and its hepatoprotective effect against CCl_4_-induced liver damage in rats, in addition to its cancer activity against colon (Caco-2) and breast (MDA-MB-231) cancer cell lines.

## 2. Materials and Methods

### 2.1. Plant Material and Extraction

The* T. porrifolius* plant material was collected from south of Lebanon and air-dried in the shade. The dried plant material was cut into small pieces and soaked in methanol for 72 hours and the extract was then filtered using Whatman no. 1 filter paper. This process was repeated twice to ensure maximal extraction. The solvent was removed under reduced pressure using a rotary evaporator (40°C, 337 mbar) and the residue was dried under vacuum (9.23% yield) and stored at −20°C until use.

### 2.2. Determination of Total Phenolic Content

Total phenolic content was estimated by the Folin-Ciocalteu colorimetric method, based on the procedure of Singleton and Rossi [12] using gallic acid (100–1000 mg/L in 80% methanol) as a standard. Briefly, 50 *μ*L of the 1 : 5 diluted and filtered extract (at an original concentration of 100 mg/mL in methanol) was mixed with 450 *μ*L of distilled water and 2.5 mL of 0.2 N Folin-Ciocalteu reagent. After 5 min, 2 mL of saturated sodium carbonate (75 g/L) was added and the mixture was incubated at 30°C for 90 minutes with intermittent shaking. The absorbance of the resulting blue-colored solution was measured at 765 nm and the total phenolic content was expressed as mg gallic acid equivalents (GAE)/g of dry weight extract.

### 2.3. Determination of Total Flavonoid Content

Total flavonoid content was determined by the aluminum chloride colorimetric method described by Chang et al. [13] using quercetin (10–100 mg/L) as a standard. Briefly, 500 *μ*L of the 1 : 20 diluted and filtered extract (at an original concentration of 100 mg/mL in methanol) was mixed with 1.5 mL of 95% methanol, 100 *μ*L of 10% aluminum chloride (AlCl_3_), 100 *μ*L of 1 M potassium acetate (CH_3_COOK), and 2.8 mL of deionized water. The mixture was incubated at room temperature for 40 minutes and the absorbance was measured at 415 nm. The total flavonoid content was expressed as quercetin equivalents mg (QE)/g dry weight extract.

### 2.4. HPLC Analysis of* T. porrifolius* Methanolic Extract

The phenolic acids and flavonoids were analyzed using Shimadzu HPLC system (Shimadzu Corp., Kyoto, Japan) consisting of LC 10-ADVP pump, SCL 10A system controller coupled with a photo-diode array detector (*SPD*-M20A), FCV-10AL low pressure gradient, Rheodyne injector (Model 7125), DGU-20A online degasser, Shim-pack VP-ODS column, (4.6 mm i.d. × 150 mm), and precolumn (10 × 4.6 mm i.d. 5 *μ*m) equipped with LC solution 1.23 SP1 software (Shimadzu, Kyoto, Japan). The column was operated at 25°C. The mobile phase consisted of water : acetic acid : methanol (10 : 2 : 88 v/v) as solvent A, and water : acetic acid : methanol (90 : 2 : 8 v/v) as solvent B at a flow rate of 1.5 mL/min. The gradient elution program was as follows: 0–15 min solvent A and 15–30 min solvent followed by washout period for 10 min and the wavelength of detection was set at 280 nm. The phenolic acids and flavonoids were identified by matching the retention time and their spectral characteristics with those of the standard compounds.

All phenolic acids and flavonoid standards (gallic acid, chlorogenic acid, vanillic acid, syringic acid, caffeic acid, ellagic acid, myricetin, quercetin, luteolin, kaempferol, and apigenin) were purchased from Sigma-Aldrich Co. (St. Louis, MO, USA). All HPLC solvents were purchased from Merck (Germany). The standard stock solutions (0.2 mg/mL) were prepared by dissolving each standard in methanol and diluted with the mobile phase in the range of 10–60 *μ*g/mL.

### 2.5. *In Vitro* Antioxidant Assays

#### 2.5.1. FRAP Assay

The FRAP value was calculated based on the method of Benzie and Strain [14] using ferrous sulphate (10–100 mM FeSO_4_·7H_2_O) as a standard. Briefly, 20 *μ*L of either the extract or different concentrations of the ferrous sulphate standard was added to 150 *μ*L of the freshly prepared and prewarmed (at 37°C) FRAP reagent (300 mM acetate buffer at a pH of 3.6, 10 mM 2,4,6-tripyridyl-s-triazine (TPTZ) in 40 mM HCl, and 20 mM ferric chloride solution in a 10 : 1 : 1 ratio). The absorbance was measured after 8 minutes in an ELISA microplate reader at 600 nm. The FRAP value (*μ*M ferric ions reduced to ferrous form per gram) of each sample was determined after subtracting the blank reading.

#### 2.5.2. DPPH Assay

The DPPH assay is based on the ability of this stable radical to react with hydrogen donors [15]. Briefly, 50 *μ*L of the extract (10, 50, and 100 *μ*g/mL) and 50 *μ*L of 0.5 mM DPPH in ethanol were added to each well and incubated in the dark for 40 minutes. The absorbance was read at 492 nm. Ascorbic acid and Trolox were used as reference compounds and the radical scavenging activity was calculated as percentage inhibition of absorbance. IC_50_, the concentration needed to reduce the initial absorbance of DPPH radical by 50%, was determined.

### 2.6. CCl_4_-Induced Hepatotoxicity Model

Male Wistar rats weighing 180–220 g (Lebanese American University stock) were housed under stable conditions of temperature (20 ± 2°C) and humidity (50 ± 5%) and an alternating cycle of light and dark (12 hr). The animals were supplied with standard laboratory rat chow diet and water. All experimental protocols were approved by the Departmental Animal Ethical Committee of the Lebanese American University, which complies with the Guide for the Care and Use of Laboratory Animals (Committee for the Update of the Guide for the Care and Use of Laboratory Animals, 2010).

Liver damage was induced with CCl_4_ in a 1 : 1 (v/v) mixture with olive oil at a dose of 1.5 mL/kg administered intraperitoneally [16]. The rats were divided into 8 groups of 6 animals each. Group I was untreated and served as normal control. Groups II and III received* T. porrifolius* methanolic extract in DMSO (50 and 250 mg/kg, i.p.) for 6 days. Group IV only received CCl_4_/olive oil (1.5 mL/kg, i.p.) for the last 3 days of treatment. Group V received DMSO (2 mL/kg) for 6 days, in combination with CCl_4_/olive oil (1.5 mL/kg, i.p) for the last 3 days of treatment (vehicle group). Groups VI, VII, and VIII were treated for 6 days with* T. porrifolius* methanolic extract in DMSO (50, 100, and 250 mg/kg, i.p), in combination with CCl_4_/olive oil (1.5 mL/kg, i.p) for the last 3 days of treatment. 48 hours after treatment, the animals were sacrificed and serum samples were tested for AST, ALT, and LDH using commercial kits (SPINREACT). Also, the liver of each animal was excised and homogenized in 0.1 M phosphate buffer (pH 7.0) containing 0.1% Triton X-100. The homogenate was centrifuged at 19,000 g for 10 minutes and the supernatant was used for the determination of total protein (Bio-Rad Protein Assay Kit II) and activity of the antioxidant enzymes CAT, SOD, and GST.

#### 2.6.1. CAT Assay

Catalase activity was assayed at 25°C according to a method described by Pedraza-Chaverrí et al. (2001) based on H_2_O_2_ disappearance [17]. The reaction between H_2_O_2_ and CAT follows a first-order kinetics as given by the equation *k* = 2.3/*t* log⁡*A*_0_/*A*, where *k* is the first-order reaction rate constant, *t* is the time over which the disappearance of H_2_O_2_ was measured (15 sec), and *A*_0_/*A* is the optical density at times 0 and 15 s, respectively. The reaction was carried out by mixing 5 *μ*L aliquots of the 1 : 40 diluted supernatant and 720 *μ*L of 30 mM H_2_O_2_ in 10 mM potassium phosphate solution. The decomposition of H_2_O_2_ by CAT present in the samples was measured at 240 nm for a period of 15 seconds. The results were expressed in k/mg protein.

#### 2.6.2. SOD Assay

SOD activity was assayed according to the method of S. Marklund and G. Marklund [18]. Briefly, 50 *μ*L of the homogenized liver supernatant was added to 2.8 mL Tris-EDTA (50 mM Tris, 1.2 mM EDTA, pH = 8.5) and 100 *μ*L of 2 mM pyrogallol at 25°C. The optical density (OD) of the mixture was read at zero and three minutes at 420 nm against the control which consisted of Tris-EDTA and pyrogallol. One unit of SOD is the amount of enzyme that inhibits the rate of autooxidation of pyrogallol by 50%. The results were calculated according to the following equations:

(1) $$\begin{array}{c} \text{Rate}\;\;\left(R\right)=\frac{\left(\text{final}\;\text{OD}-\text{initial}\;\text{OD}\right)}{3},\\ \%\;\;\text{inhibition}=\left[\frac{\left(R_{\text{control}}\;-\;R\right)}{R_{\text{control}}}\right]\times 100,\\ \text{Enzyme}\;\text{unit}\;\left(U\right)=\left(\frac{\%\;\;\text{inhibition}}{50}\right)\times \text{dilution}\;\text{factor}. \end{array}$$

#### 2.6.3. GST Assay

GST activity was determined according to the method of Habig et al. [19]. This procedure is based on the conjugation of glutathione (GSH) to 1-chloro-2,4-dinitrobenzene (CDNB) as a substrate. Briefly, 600 *μ*L of the liver homogenate supernatant fraction was added to 2.2 mL of 0.1 M potassium phosphate buffer (pH 6.5), 100 *μ*L of 30 mM CDNB, and 100 *μ*L of 30 mM GSH. After adding CDNB the change in absorbance at 340 nm was determined at 37°C as a function of time and the activity of GST was expressed in nmol of GSH-CDNB conjugates formed/min/mg protein using an extinction coefficient of 9.6 mM^−1 ^cm^−1^.

### 2.7. Cell Culture and Treatment

MDA-MB-231 (human breast adenocarcinoma) and Caco-2 (human colorectal adenocarcinoma) cell lines were used to assess cytotoxicity and antiproliferation effects. Both cell lines were maintained in Dulbecco's modified Eagle's medium (DMEM) supplemented with 10% v/v heat-inactivated fetal bovine serum (FBS), 100 *μ*g/mL streptomycin, and 100 U/mL penicillin. Cells were incubated at 37°C in a humidified atmosphere of 5% CO_2_.

#### 2.7.1. Cytotoxicity Assay

Cytotoxicity of the* T. porrifolius* methanolic extract (5, 10, 25, 50, and 100 *μ*g/mL) on the cell lines was assayed at 24 and 48 hours using the Trypan Blue exclusion method. Cells were trypsinized, diluted in 0.4% Trypan Blue, and counted in a hemocytometer chamber. Cells were plated in triplicate and experiments were repeated three times.

#### 2.7.2. Proliferation Assay

The effect of the* T. porrifolius* methanolic extract (5, 10, 25, 50, and 100 *μ*g/mL) on cell proliferation was measured at 24 and 48 hours using the cell proliferation reagent WST-1 (Roche Applied Science, Penzberg, Germany), a tetrazolium salt which is cleaved by mitochondrial dehydrogenases in metabolically active cells. The intensity of produced formazan was measured at 440 nm using a microplate ELISA reader. Cells were plated in triplicate and experiments were repeated three times.

### 2.8. Statistical Analysis

Data was analyzed for statistical significance using one-way analysis of variance (ANOVA). Values of the different tested parameters within each group are presented as mean ± SEM. All data were analyzed with the statistical package SPSS 18, and differences between groups were considered statistically significant if *P* < 0.05.

## 3. Results

### 3.1. Total Phenolic and Flavonoid Contents

Total phenolic content of the methanolic extract of* T. porrifolius* was estimated by the Folin-Ciocalteu colorimetric method using gallic acid to generate the standard curve and was determined to be 37.0 ± 1.4 mg GAE/g dry weight. Total flavonoid content was estimated by the aluminum chloride colorimetric method using quercetin to generate the standard curve and was determined to be 16.6 ± 0.42 mg QE/g dry weight.

### 3.2. *In Vitro* Antioxidant Activity

The* in vitro* antioxidant activity of the* T. porrifolius* methanolic extract was determined by two methods: the FRAP assay and the DPPH free radical scavenging assay. Results shown in Table 1 reveal that the FRAP value and IC_50_ value of DPPH were 659 *μ*mol Fe^2+^/g and 15.2 *μ*g/mL, respectively.

### 3.3. HPLC Analysis of* T. porrifolius* Methanolic Extract

**Figure 1:**
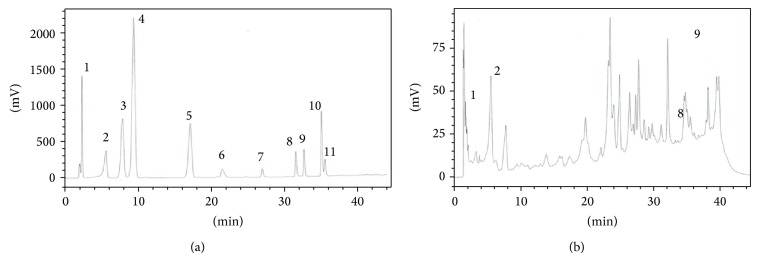
(a) HPLC chromatogram of a standard mixture of phenolic acids and flavonoids. Peaks: 1 = gallic acid; 2 = chlorogenic acid; 3 = vanillic acid; 4 = syringic acid; 5 = caffeic acid; 6 = ellagic acid; 7 = myricetin; 8 = quercetin; 9 = luteolin; 10 = kaempferol; 11 = apigenin. (b) HPLC chromatogram of* Tragopogon porrifolius* methanolic extract.

The* T. porrifolius* methanolic extract was subjected to HPLC analysis and the results are displayed in Figure 1. The HPLC chromatogram showed several peaks, and only four peaks matched with the standards with retention times 2.13, 5.88, 31.35, and 32.4 min were identified as gallic acid (1.60 *μ*g/mg), chlorogenic acid (13.9 *μ*g/mg), quercetin (15.4 *μ*g/mg), and luteolin (35.34 *μ*g/mg), respectively, by comparing their retention times and UV spectra.

### 3.4. Hepatoprotective Activity

Treatment of normal rats with* T. porrifolius* methanolic extract (50 and 250 mg/kg body weight) did not significantly affect the activities of AST, ALT, and LDH (Table 2). As expected, the groups subjected to liver damage with CCl_4_/olive oil showed tremendous increases in the levels of these enzymes (Table 2). However, pretreatment with the extract (50, 100, and 250 mg/kg) exhibited a significant hepatoprotective capacity as compared to the control (group V) that received DMSO + CCl_4_/olive oil. The aforementioned doses caused significant reduction in levels of AST by 26.9, 40.2, and 51.7%, ALT by 17.9, 31.8, and 50.7%, and LDH by 30.5, 48.8, and 61.1%, respectively.

The* in vivo* antioxidant activity of* T. porrifolius* methanolic extract was determined by evaluating the activities of CAT, GST, and SOD in the livers of both normal and CCl_4_-treated animals (Table 3). Treatment of normal rats with the lowest and highest doses of the extract increased the activities of these enzymes, with significance reached at 250 mg/kg body weight. CCl_4_ treatment significantly lowered the activities of CAT, GST, and SOD as compared to the normal group. However, groups pretreated with the extract showed a significant dose-dependent increase in the activities of these enzymes which were restored to normal at 250 mg/kg body weight dose.

### 3.5. Effects of* T. porrifolius* Methanolic Extract on Cytotoxicity and Cell Proliferation

**Figure 2:**
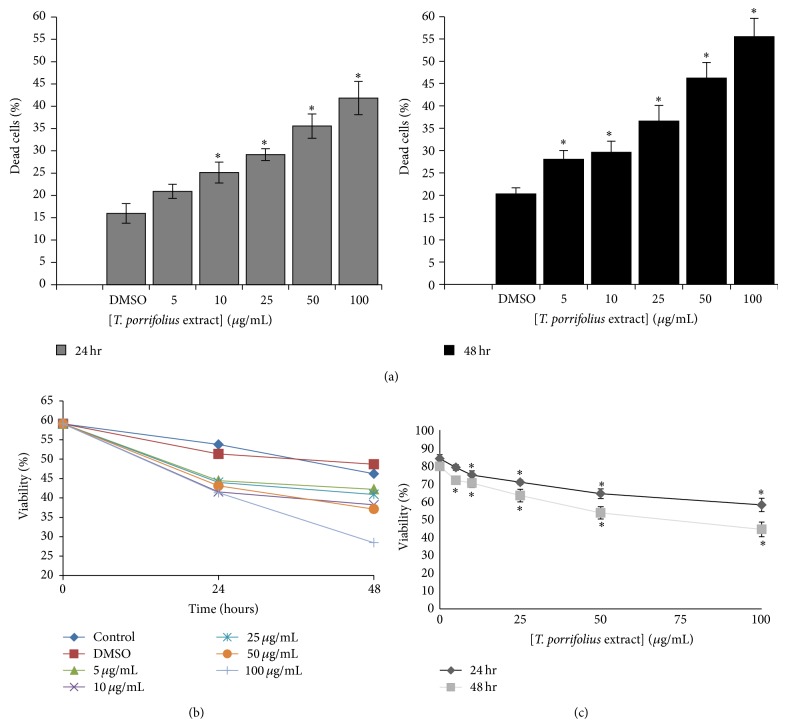
Cytotoxicity of* T. porrifolius* methanolic extract on MDA-MB-231 cells at 24 and 48 hours of treatment. (a and b) Effect of the extract on cell viability. (c) Dose response curve of the extract. ^∗^ *P* < 0.02 with respect to the group that received only DMSO.

**Figure 3:**
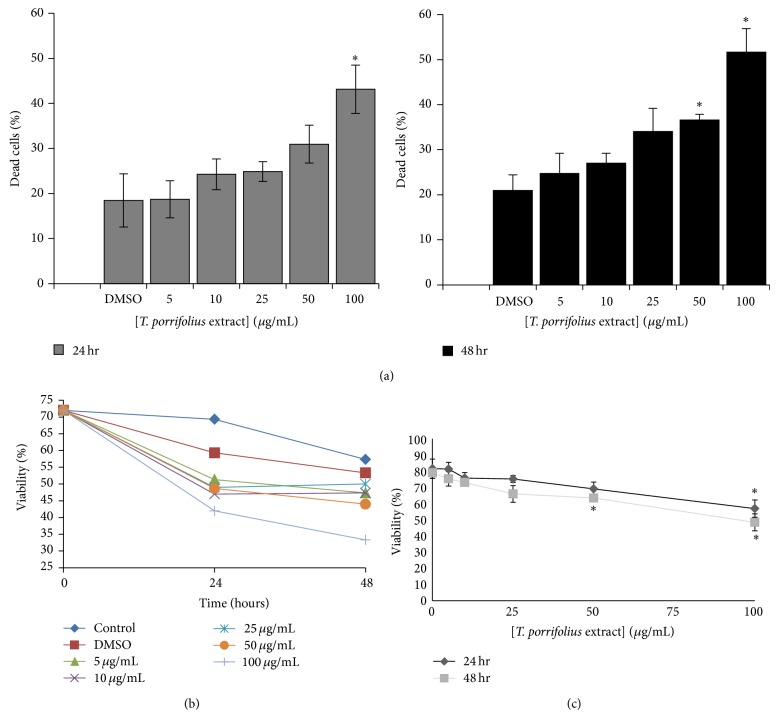
Cytotoxicity of* T. porrifolius* methanolic extract on Caco-2 cells at 24 and 48 hours of treatment. (a and b) Effect of the extract on cell viability. (c) Dose response curve of the extract. ^∗^ *P* < 0.05 with respect to the group that received only DMSO.

The cytotoxic effect of the* T. porrifolius* methanolic extract was also examined. In both MDA-MB-231 (Figure 2) and Caco-2 (Figure 3), the cells showed a decrease in cell viability that is both time- and dose-dependent. The MDA-MB-231 cells showed between 25% and 35% increase in dead cells after treatment with 100 *μ*g/mL of the extract at 24 and 48 hours (Figure 2(a)). Similarly, the Caco-2 cells showed between 20% and 30% increase in dead cells after treatment with 100 *μ*g/mL of the extract at 24 and 48 hours (Figure 3(a)). In both cell lines, treating the cells for 48 hours and at a concentration of 100 *μ*g/mL showed the optimum activity with around 35% decrease in cell viability (Figures 2(b), 2(c), 3(b), and 3(c)).

**Figure 4:**
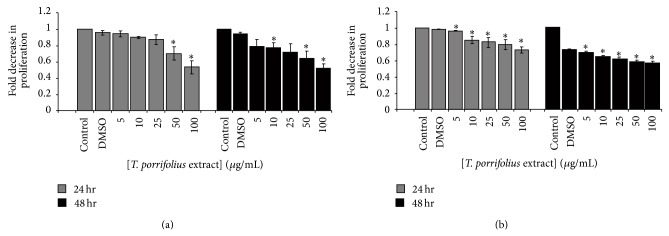
The effect of* T. porrifolius* on proliferation of MDA-MB-231 (a) and Caco-2 (b) cell lines in the presence of increasing concentration of the methanolic extract at 24 and 48 hours of treatment. ^∗^ *P* < 0.05 with respect to the group that received only DMSO.

The effect of the* T. porrifolius* methanolic extract on the proliferation of the MDA-MB-231 and Caco-2 cell lines was evaluated using the WST-1 proliferation assay. The results, shown in Figure 4, demonstrate a dose-dependent decrease in proliferation. The optimal concentration of 100 *μ*g/mL was again used and, similarly to the viability results, reduced proliferation by almost 50%. The effect on Caco-2 cells was less pronounced, consistently with the viability data.

## 4. Discussion

Reactive oxygen species (ROS) have been associated with many degenerative diseases and are considered potential carcinogens. These oxygen-derived free radicals can induce injury to nuclear DNA, facilitating mutagenesis and leading to cancer initiation and progression [4]. Antioxidants provide protection to cellular systems from the harmful effects of excessive oxidation and consequently contribute to the inhibition of many diseases including cardiovascular diseases and cancer [20].

The antioxidant capacity of the* T. porrifolius* extract was tested using* in vitro* and* in vivo* assays. In the FRAP assay, the extract had a value of 659 *μ*mol Fe^2+^/g dry weight which was fairly comparable to that of ascorbic acid, 889 *μ*mol Fe^2+^/g. Song et al. [21] reported the antioxidant capacities of 56 Chinese medicinal plants where the FRAP values ranged from 0.15 *μ*mol Fe^2+^/g to 856.9 *μ*mol Fe^2+^/g. Therefore,* T. porrifolius* methanolic extract can be considered to have a relatively high antioxidant capacity. Similarly, the DPPH radical scavenging assay revealed high antioxidant activity (IC_50_ = 15.18 *μ*g/mL) that is also comparable to that of ascorbic acid (IC_50_ = 9.13 *μ*g/mL).

Polyphenols are capable of scavenging free radicals by donating a hydrogen atom and are thus considered efficient antioxidants in a wide range of oxidation systems [20]. Total phenolic content of the* T. porrifolius* methanolic extract was calculated to be 36.9 mg GAE/g of plant extract dry weight. Studies by Song et al. [21] and Tawaha et al. [22] showed a range of total phenolic content between 0.12 and 59.6 mg GAE/g of plant extract. According to Tawaha et al. [22], any value greater than 20 mg GAE/g of plant extract was considered to be remarkably high, thus indicating that the* T. porrifolius* methanolic extract is a rich source of polyphenols. Flavonoids are known to have beneficial effects on a variety of diseases, including cardiovascular diseases and cancer [23].* T. porrifolius* flavonoid content (16.6 mg QE/g dry weight) is considered relatively high when compared to some Mediterranean dietary plants [24] reflecting the potential benefits of the plant.

Antioxidant enzymes constitute the first line of defense against oxidative stress and damage caused by free radicals [25]. In this study,* in vivo* data showed that animals treated with CCl_4_ exhibited a substantial (*P* < 0.001) decrease in the levels of liver antioxidant enzymes as compared to control animals, which is consistent with previous reports in the literature [26]. Treatment with* T. porrifolius* methanolic extract appeared to have a positive impact on the level of liver antioxidant enzymes when given to normal rats or to rats subjected to liver damage. The effect was most remarkable on the CAT levels where normal animals receiving the extract (50 and 250 mg/kg, groups II and III) exhibited 22.6 and 78.2% increase in the level of this enzyme, respectively. Also, in the CCl_4_-treated groups (VI, VII, and VIII), the extract raised the CAT levels by 72, 124, and 222%, respectively, compared to the control group. The effects of the extract were less pronounced on the levels of SOD and GST enzymes whereby group II increased SOD and GST levels by only 13 and 16% and group III by only 27 and 29%. Similarly, the extract increased SOD levels by 23, 37, and 68% in the CCl_4_-treated groups (VI, VII, and VIII), respectively. As for GST, only the 100 and 250 mg/kg body weight doses resulted in an increment of 36 and 149%, respectively, as compared to the control group. HPLC analysis in this study revealed the presence of quercetin, luteolin, gallic acid, and chlorogenic acid, which is consistent with previous reports [27–31]. Gallic acid and chlorogenic acid have been reported to possess antioxidant [32–34] and hepatoprotective activity against CCl_4_-induced liver damage [35, 36]. Similarly, quercetin and luteolin have also been shown to possess antioxidant activity [37, 38].

The effect of the* T. porrifolius* methanolic extract on liver enzymes (ALT, AST, and LDH) was assessed to evaluate any potential hepatic damage. In normal rats, the extract did not cause any significant change in the level of these enzymes. However, in the CCl_4_-treated groups, all used doses of the extract were able to protect the liver and reverse the enzyme levels to normal especially with the 250 mg/kg dose. Jadhav et al. [39] reported similar hepatoprotection against CCl_4_ while using silymarin (a reference drug) at a concentration of 200 mg/kg, an effect analogous to the 250 mg/kg dose of* T. porrifolius* methanolic extract.

The anticancer effect of the methanolic extract of* T. porrifolius* was investigated* in vitro* on breast (MDA-MB-231) and colorectal (Caco-2) adenocarcinoma cell lines. The present data showed an antiproliferative effect on both cell lines in a time- and dose-dependent manner. In the WST-1 proliferation assay, a statistically significant decrease in proliferation was recorded by the highest dose of the extract (100 *μ*g/mL). This effect was slightly greater in the MDA-MB-231 cells with maximum inhibition of around 40% observed at 24 hrs after treatment. The Trypan Blue exclusion method showed that the extract increases cell death at this concentration. Hence, the decrease in metabolically active cells as revealed in the WST-1 assay might be due to a positive effect of the extract on cell death. The anticancer property of* T. porrifolius* could be attributed to its relatively high flavonoid component and/or its antioxidant effect or a different mechanism. Antioxidants can decrease oxidative stress-induced carcinogenesis by inhibiting ROS generation or through overexpressing antioxidant enzymes.

In addition to their antioxidant activity, flavonoids are also known to produce antitumor activity through inhibition of proliferation, metastasis and invasive effects, induction of apoptosis, suppression of protein tyrosine kinase activity, and antiangiogenesis [40]. The identified flavonoids and phenolic acids have been reported to possess anticancer effects against different types of cancer cells. For example, chlorogenic acid was shown to inhibit proliferation of murine melanoma, human adenocarcinoma, and uterine carcinoma cells [41, 42]. Kuntz et al. [43] reported that luteolin and quercetin displayed antiproliferative effects against colon cancer cell lines HT-29 and Caco-2. Luteolin has also been found to be effective against various types of cancer cells [44, 45]. Inhibition of human colon and breast cancer cells proliferation by quercetin has been well cited in the literature [46–50]. Gallic acid was also reported to inhibit proliferation [51] and induce differentiation [52] and cell cycle delay [53] in Caco-2 cells. Additionally, gallic acid inhibited the activation of NF*κ*B resulting in inhibition of target genes involved in metastasis, antiapoptosis, and angiogenesis [54].

In conclusion, the* Tragopogon porrifolius* methanolic extract was shown to possess antioxidant, hepatoprotective, and anticancer potentials. The antioxidant activity was demonstrated both* in vitro* and* in vivo*. This effect may be attributed to the relatively high contents of phenols and flavonoids. The extract at all doses used showed no negative effects on liver enzymes in normal rats and protected the liver against CCl_4_-induced toxicity. The anticancer activity against MDA-MB-231 and Caco-2 cell lines was time- and dose-dependent and was mediated through inhibition of cell proliferation and increased cell death. Future work is needed to characterize the phenolic and flavonoid content as well as the biologically active compounds.

## Figures and Tables

**Table 1:** *In vitro* antioxidant activity of *T*. *porrifolius* methanolic extract in comparison to ascorbic acid and Trolox, as calculated by the FRAP and DPPH assays. Values denote mean ± SEM (*n* = 5).

	FRAP value (*µ*mol Fe^2+^/g)	DPPH assay-IC_50_ (*µ*g/mL)
*T*. *porrifolius* methanolic extract	659.57 ± 13.77	15.18
Ascorbic acid	889.27 ± 17.13	9.13
Trolox	1349.86 ± 53.41	6.82

**Table 2:** Effect of *T*. *porrifolius* methanolic extract on the activities of liver antioxidant enzymes. Values denote mean ± SEM (*n* = 6).

Group		CAT (k/mg)	GST (nmol/min/mg)	SOD (units)
I	Normal (no treatment)	4.77 ± 0.54	7.37 ± 0.52	13.44 ± 0.60
II	*T*. *porrifolius* 50 mg/kg	5.85 ± 0.81	8.51 ± 0.73	15.25 ± 0.68
III	*T*. *porrifolius* 250 mg/kg	8.51 ± 0.54^a^	9.54 ± 0.65^a^	17.00 ± 0.56^a^
IV	CCl_4_/olive oil	1.05 ± 0.12^a^	0.92 ± 0.13^a^	6.33 ± 0.74^a^
V	DMSO + CCl_4_/olive oil	1.43 ± 0.11^a^	2.35 ± 0.51^a^	8.38 ± 0.52^a^
VI	*T*. *porrifolius* 50 mg/kg + CCl_4_/olive oil	2.46 ± 0.34^ab^	1.64 ± 0.16^a^	10.32 ± 0.91^a^
VII	*T*. *porrifolius* 100 mg/kg + CCl_4_/olive oil	3.19 ± 0.35^ab^	3.19 ± 0.41^a^	10.91 ± 0.63^ab^
VIII	*T*. *porrifolius* 250 mg/kg + CCl_4_	4.61 ± 0.27^b^	5.86 ± 0.65^b^	14.11 ± 0.73^b^

^a^ *P* < 0.05 with respect to the normal group (no treatment).

^b^ *P* < 0.001 with respect to the vehicle group (DMSO + CCl_4_/olive oil).

**Table 3:** Effect of *T*. *porrifolius* methanolic extract on the activities of liver function enzymes in serum. Values denote mean ± SEM (*n* = 6).

Group		AST (U/L)	ALT (U/L)	LDH (U/L)
I	Normal (no treatment)	61.83 ± 2.44	33.44 ± 2.63	312.74 ± 53.27
II	*T*. *porrifolius* 50 mg/kg	60.76 ± 0.86	35.58 ± 1.20	315.2 ± 16.74
III	*T*. *porrifolius* 250 mg/kg	67.81 ± 1.54	38.35 ± 2.45	369.37 ± 27.17
IV	CCl_4_/olive oil	137.57 ± 9.03	66.30 ± 4.10	798.40 ± 53.57
V	DMSO + CCl_4_/olive oil	122.89 ± 14.22	58.62 ± 4.24	727.53 ± 96.12
VI	*T*. *porrifolius* 50 mg/kg + CCl_4_/olive oil	89.83 ± 8.20^∗^	48.12 ± 5.71	505.63 ± 62.30^∗^
VII	*T*. *porrifolius* 100 mg/kg + CCl_4_/olive oil	73.5 ± 1.86^∗^	39.96 ± 2.63^∗^	372.38 ± 28.12^∗^
VIII	*T*. *porrifolius* 250 mg/kg + CCl_4_	59.30 ± 5.82^∗^	28.87 ± 5.65^∗^	282.37 ± 17.97^∗^

^∗^ *P* < 0.05 with respect to the group that received DMSO and CCl_4_.

## References

[1] Halliwell B., Gutteridge J. (2006). Antioxidant defenses endogenous and diet derived. *Free Radicals in Biology and Medicine*.

[2] Waris G., Ahsan H. (2006). Reactive oxygen species: role in the development of cancer and various chronic conditions. *Journal of Carcinogenesis*.

[3] Evans M. D., Dizdaroglu M., Cooke M. S. (2004). Oxidative DNA damage and disease: induction, repair and significance. *Mutation Research*.

[4] Manda G., Nechifor M. T., Neagu T.-M. (2009). Reactive oxygen species, cancer and anti-cancer therapies. *Current Chemical Biology*.

[5] World Health Organization

[6] Fabricant D. S., Farnsworth N. R. (2001). The value of plants used in traditional medicine for drug discovery. *Environmental Health Perspectives*.

[7] Ma J. K.-C., Chikwamba R., Sparrow P., Fischer R., Mahoney R., Twyman R. M. (2005). Plant-derived pharmaceuticals—the road forward. *Trends in Plant Science*.

[8] Gupta K., Talwar G., Jain V., Dhawan K., Jain S. (2000). Salad crops root, bulb, and tuber crops. *Encyclopaedia of Food Science*.

[9] Formisano C., Rigano D., Senatore F., Bruno M., Rosselli S. (2010). Volatile constituents of the aerial parts of white salsify (*Tragopogon porrifolius* L., Asteraceae). *Natural Product Research*.

[10] Zeeni N., Daher C. F., Saab L., Mroueh M. (2014). *Tragopogon porrifolius* improves serum lipid profile and increases short-term satiety in rats. *Appetite*.

[11] Hariri E. H., El Asmar M., Demirdjian S. A., Daher C. F., Mroueh M. A. (2013). Anti-inflammatory effect of the methanol, ethyl acetate and chloroform extracts of *Tragopogon porrifolius* aerial parts. *Planta Medica*.

[12] Singleton V., Rossi J. A. (1965). Colorimetry of total phenolics with phosphomolybdic-phosphotungstic acid reagents. *American Journal of Enology and Viticulture*.

[13] Chang C.-C., Yang M.-H., Wen H.-M., Chern J.-C. (2002). Estimation of total flavonoid content in propolis by two complementary colometric methods. *Journal of Food and Drug Analysis*.

[14] Benzie I. F. F., Strain J. J. (1996). The ferric reducing ability of plasma (FRAP) as a measure of ‘antioxidant power’: the FRAP assay. *Analytical Biochemistry*.

[15] Chu W. L., Lim Y. W., Radhakrishnan A. K., Lim P. E. (2010). Protective effect of aqueous extract from *Spirulina platensis* against cell death induced by free radicals. *BMC Complementary and Alternative Medicine*.

[16] Coballase-Urrutia E., Pedraza-Chaverri J., Cárdenas-Rodríguez N. (2011). Hepatoprotective effect of acetonic and methanolic extracts of *Heterotheca* inuloides against CCl_4_-induced toxicity in rats. *Experimental and Toxicologic Pathology*.

[17] Pedraza-Chaverrí J., De los Ángeles Granados-Silvestre M., Medina-Campos O. N., Maldonado P. D., Olivares-Corichi I. M., Ibarra-Rubio M. E. (2001). Post-transcriptional control of catalase expression in garlic-treated rats. *Molecular and Cellular Biochemistry*.

[18] Marklund S., Marklund G. (1974). Involvement of the superoxide anion radical in the autoxidation of pyrogallol and a convenient assay for superoxide dismutase. *European Journal of Biochemistry*.

[19] Habig W. H., Pabst M. J., Jakoby W. B. (1974). Glutathione S-transferases. The first enzymatic step in mercapturic acid formation. *Journal of Biological Chemistry*.

[20] Duthie G. G., Duthie S. J., Kyle J. A. M. (2000). Plant polyphenols in cancer and heart disease: implications as nutritional antioxidants. *Nutrition Research Reviews*.

[21] Song F.-L., Gan R.-Y., Zhang Y., Xiao Q., Kuang L., Li H.-B. (2010). Total phenolic contents and antioxidant capacities of selected Chinese medicinal plants. *International Journal of Molecular Sciences*.

[22] Tawaha K., Alali F. Q., Gharaibeh M., Mohammad M., El-Elimat T. (2007). Antioxidant activity and total phenolic content of selected Jordanian plant species. *Food Chemistry*.

[23] Hou D.-X., Kumamoto T. (2010). Flavonoids as protein kinase inhibitors for cancer chemoprevention: direct binding and molecular modeling. *Antioxidants and Redox Signaling*.

[24] Conforti F., Sosa S., Marrelli M. (2008). In vivo anti-inflammatory and in vitro antioxidant activities of Mediterranean dietary plants. *Journal of Ethnopharmacology*.

[25] Agbafor K. N., Nwachukwu N. (2011). Phytochemical analysis and antioxidant property of leaf extracts of *Vitex doniana* and *Mucuna pruriens*. *Biochemistry Research International*.

[26] Ganie S. A., Haq E., Masood A., Zargar M. A. (2010). Amelioration of carbon tetrachloride induced oxidative stress in kidney and lung tissues by ethanolic rhizome extract of *Podophyllum hexandrum* in Wistar rats. *Journal of Medicinal Plants Research*.

[27] Bohm B. A., Stuessy T. F. (2001). Flavonoids of Lactuceae. *Flavonoids of the Sunflower Family (Asteraceae)*.

[28] Kroschewsky J. R., Mabry T. J., Markham K. R., Alston R. E. (1969). Flavonoids from the genus *Tragopogon* (compositae). *Phytochemistry*.

[29] Sareedenchai V., Ganzera M., Ellmerer E. P., Lohwasser U., Zidorn C. (2009). Phenolic compounds from *Tragopogon porrifolius* L.. *Biochemical Systematics and Ecology*.

[30] Spina M., Cuccioloni M., Sparapani L. (2008). Comparative evaluation of flavonoid content in assessing quality of wild and cultivated vegetables for human consumption. *Journal of the Science of Food and Agriculture*.

[31] Zidorn C., Ellmerer E. P., Sturm S., Stuppner H. (2003). Tyrolobibenzyls E and F from *Scorzonera humilis* and distribution of caffeic acid derivatives, lignans and tyrolobibenzyls in European taxa of the subtribe Scorzonerinae (Lactuceae, Asteraceae). *Phytochemistry*.

[32] Sroka Z., Cisowski W. (2003). Hydrogen peroxide scavenging, antioxidant and anti-radical activity of some phenolic acids. *Food and Chemical Toxicology*.

[33] Sato Y., Itagaki S., Kurokawa T. (2011). In vitro and in vivo antioxidant properties of chlorogenic acid and caffeic acid. *International Journal of Pharmaceutics*.

[34] Kweon M.-H., Hwang H.-J., Sung H.-C. (2001). Identification and antioxidant activity of novel chlorogenic acid derivatives from bamboo (*Phyllostachys edulis*). *Journal of Agricultural and Food Chemistry*.

[35] Shi H., Dong L., Bai Y., Zhao J., Zhang Y., Zhang L. (2009). Chlorogenic acid against carbon tetrachloride-induced liver fibrosis in rats. *European Journal of Pharmacology*.

[36] Tung Y.-T., Wu J.-H., Huang C.-C. (2009). Protective effect of *Acacia confusa* bark extract and its active compound gallic acid against carbon tetrachloride-induced chronic liver injury in rats. *Food and Chemical Toxicology*.

[37] Rao Y. K., Geethangili M., Fang S.-H., Tzeng Y.-M. (2007). Antioxidant and cytotoxic activities of naturally occurring phenolic and related compounds: a comparative study. *Food and Chemical Toxicology*.

[38] Seelinger G., Merfort I., Schempp C. M. (2008). Anti-oxidant, anti-inflammatory and anti-allergic activities of luteolin. *Planta Medica*.

[39] Jadhav V. B., Thakare V. N., Suralkar A. A., Deshpande A. D., Naik S. R. (2010). Hepatoprotective activity of *Luffa acutangula* against CCl4 and rifampicin induced liver toxicity in rats: a biochemical and histopathological evaluation. *Indian Journal of Experimental Biology*.

[40] Kanadaswami C., Lee L.-T., Lee P.-P. H. (2005). The antitumor activities of flavonoids. *In Vivo*.

[41] Abe F., Nagao T., Okabe H. (2002). Antiproliferative constituents in plants 9. Aerial parts of *Lippia dulcis* and *Lippia canescens*. *Biological and Pharmaceutical Bulletin*.

[42] Nagao T., Abe F., Okabe H. (2001). Antiproliferative constituents in the plants 7. Leaves of *Clerodendron bungei* and leaves and bark of *C. trichotomum*. *Biological and Pharmaceutical Bulletin*.

[43] Kuntz S., Wenzel U., Daniel H. (1999). Comparative analysis of the effects of flavonoids on proliferation, cytotoxicity, and apoptosis in human colon cancer cell lines. *European Journal of Nutrition*.

[44] Leonardi T., Vanamala J., Taddeo S. S. (2010). Apigenin and naringenin suppress colon carcinogenesis through the aberrant crypt stage in azoxymethane-treated rats. *Experimental Biology and Medicine*.

[45] Lee E.-J., Oh S.-Y., Sung M.-K. (2012). Luteolin exerts anti-tumor activity through the suppression of epidermal growth factor receptor-mediated pathway in MDA-MB-231 ER-negative breast cancer cells. *Food and Chemical Toxicology*.

[46] Gibellini L., Pinti M., Nasi M. (2010). Interfering with ROS metabolism in cancer cells: the potential role of quercetin. *Cancers*.

[47] Chien S.-Y., Wu Y.-C., Chung J.-G. (2009). Quercetin-induced apoptosis acts through mitochondrial- and caspase-3-dependent pathways in human breast cancer MDA-MB-231 cells. *Human and Experimental Toxicology*.

[48] Xiao X., Shi D., Liu L. (2011). Quercetin suppresses cyclooxygenase-2 expression and angiogenesis through inactivation of P300 signaling. *PLoS ONE*.

[49] Yang J., Liu R. H. (2009). Synergistic effect of apple extracts and quercetin 3-*β*-d-glucoside combination on antiproliferative activity in MCF-7 human breast cancer cells in vitro. *Journal of Agricultural and Food Chemistry*.

[50] Zhang H., Zhang M., Yu L., Zhao Y., He N., Yang X. (2012). Antitumor activities of quercetin and quercetin-5′,8-disulfonate in human colon and breast cancer cell lines. *Food and Chemical Toxicology*.

[51] Forester S. C., Waterhouse A. L. (2010). Gut metabolites of anthocyanins, gallic acid, 3-*O*-methylgallic acid, and 2,4,6-trihydroxybenzaldehyde, inhibit cell proliferation of Caco-2 cells. *Journal of Agricultural and Food Chemistry*.

[52] Lea M. A., Ibeh C., Han L., Desbordes C. (2010). Inhibition of growth and induction of differentiation markers by polyphenolic molecules and histone deacetylase inhibitors in colon cancer cells. *Anticancer Research*.

[53] Salucci M., Stivala L. A., Maiani G., Bugianesi R., Vannini V. (2002). Flavonoids uptake and their effect on cell cycle of human colon adenocarcinoma cells (Caco2). *British Journal of Cancer*.

[54] García-Rivera D., Delgado R., Bougarne N., Haegeman G., Berghe W. V. (2011). Gallic acid indanone and mangiferin xanthone are strong determinants of immunosuppressive anti-tumour effects of *Mangifera indica* L. bark in MDA-MB231 breast cancer cells. *Cancer Letters*.

